# Therapeutic potential of the renin angiotensin system in ischaemic stroke

**DOI:** 10.1186/s13231-016-0022-1

**Published:** 2016-10-07

**Authors:** Mariana Moreira Coutinho Arroja, Emma Reid, Christopher McCabe

**Affiliations:** Institute of Neuroscience and Psychology, College of Medical, Veterinary and Life Sciences, University of Glasgow, Garscube Estate, Glasgow, G61 1QH UK

**Keywords:** Acute ischaemic stroke (AIS), Renin angiotensin system (RAS), Angiotensin II (Ang II), Angiotensin-(1–7) (Ang-(1–7)), AT_1_R blockers, AT_2_R agonists, MasR agonists

## Abstract

The renin angiotensin system (RAS) consists of the systemic hormone system, critically involved in regulation and homeostasis of normal physiological functions [i.e. blood pressure (BP), blood volume regulation], and an independent brain RAS, which is involved in the regulation of many functions such as memory, central control of BP and metabolic functions. In general terms, the RAS consists of two opposing axes; the ‘classical axis’ mediated primarily by Angiotensin II (Ang II), and the ‘alternative axis’ mediated mainly by Angiotensin-(1–7) (Ang-(1–7)). An imbalance of these two opposing axes is thought to exist between genders and is thought to contribute to the pathology of cardiovascular conditions such as hypertension, a stroke co-morbidity. Ischaemic stroke pathophysiology has been shown to be influenced by components of the RAS with specific RAS receptor antagonists and agonists improving outcome in experimental models of stroke. Manipulation of the two opposing axes following acute ischaemic stroke may provide an opportunity for protection of the neurovascular unit, particularly in the presence of pre-existing co-morbidities where the balance may be shifted. In the present review we will give an overview of the experimental stroke studies that have investigated pharmacological interventions of the RAS.

## Background

In the UK, stroke is the fourth leading cause of death and one of the largest contributors towards long-term disability, affecting approximately 152,000 people every year (https://www.stroke.org.uk; State of the Nation 2015) [1]. In the past 40 years the number of stroke fatalities has been decreasing, however, it is estimated that over two-thirds of stroke survivors require daily medical care and over half are left disabled [2], resulting in an annual cost of nearly £4 billion and accounting for approximately 4–6 % of total NHS expenditure [3].

Recombinant tissue plasminogen activator (rt-PA; Alteplase), is the only thrombolytic treatment currently available for acute ischaemic stroke (AIS). It acts by breaking down the clot or thrombus obstructing the cerebral vessel, thus, re-establishing blood flow. However, it has a narrow therapeutic time window of 4.5 h from stroke onset, resulting in only 2–5 % of ischaemic strokes being treated globally, and can have detrimental side effects, including haemorrhage [4]. Recent results from a number of randomised clinical trials of mechanical thrombectomy have demonstrated efficacy for this intervention up to 6 h after stroke onset [5]. The positive results from these trials have reinvigorated the stroke community and open up new possibilities for adjunctive protective strategies.

Failure to translate effective therapeutic strategies from the ‘bench to bedside’ may partly be attributed to the use of animal models that do not incorporate non-modifiable risk factors such as gender and many of the stroke co-morbidities observed in the clinical stroke population, such as hypertension, diabetes, obesity, etc. For instance, hypertension is the single most important modifiable risk factor for stroke, acting as a contributing factor in over 75 % of first time stroke patients [6] with hypertension during acute stroke is associated with poorer clinical outcome [7].

The renin angiotensin system (RAS), a peptide hormone system intrinsically involved in blood pressure regulation and blood volume homeostasis in the circulation, has been shown to be present as a local paracrine system in the brain [8]. The RAS is reported to be involved in the pathology of AIS and its risk factors [8, 9], therefore, emerging as a potential therapeutic target. This review discusses the therapeutic potential of the RAS following AIS, emphasising the importance of cerebral RAS receptor targeting and its relevance in the presence of known stroke risk factors.

### Brain RAS: classical and alternative axis

**Fig. 1:**
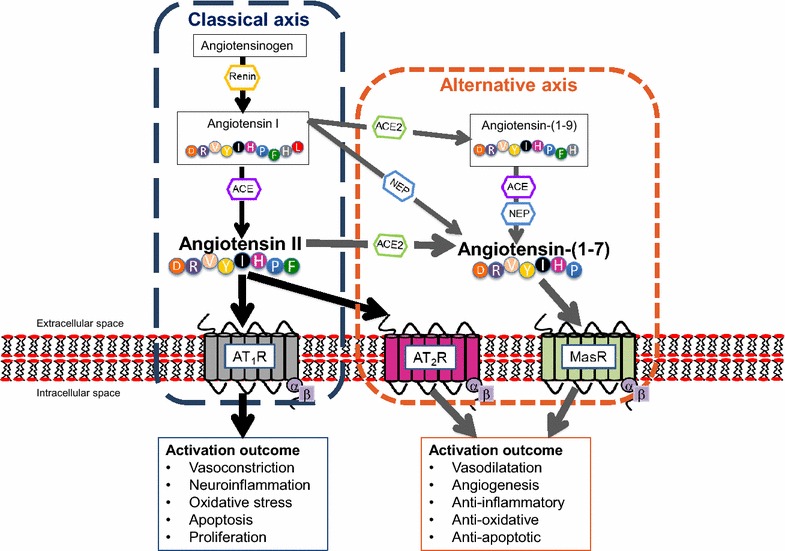
Simplified overview of the renin angiotensin system (RAS). Angiotensinogen is cleaved by renin generating Angiotensin I (Ang I) which is then formed into Angiotensin II (Ang II) via the actions of angiotensin converting enzyme (ACE). Ang II preferentially binds to Angiotensin II type I receptor (AT_1_R) (‘classical axis’) inducing vasoconstriction, inflammation, oxidative stress, apoptosis and cell proliferation. Ang II can also activate the Angiotensin II type II (AT_2_R) and is metabolised by angiotensin converting enzyme 2 (ACE2) to generate Angiotensin-(1–7). Ang-(1–7) activates the Mas receptor (MasR). Ang-(1–7) can be formed by the actions of ACE or neprilysin (NEP) on Angiotensin-(1–9) or Angiotensin I. AT_2_R and ACE2/Ang-(1–7)/MasR form the ‘alternative axis’ and its activation is thought to counteract the detrimental effects induced by AT_1_R by leading to vasodilation, angiogenesis and preventing inflammation, oxidative stress and apoptosis

In the circulation, a drop in blood pressure (systemic hypotension) and/or blood volume results in juxtaglomerular cells within the kidneys to release renin (protease) whereas increased blood pressure (hypertension) inhibits renin release under normal circumstances. Circulating angiotensinogen is hydrolysed by renin to produce Angiotensin I (Ang I), which is then further converted by angiotensin converting enzyme (ACE) to generate biological active octapeptide, Ang II (Fig. 1). Ang II, a potent vasoconstrictor, acts by stimulating Ang II type 1 and type 2 receptors (AT_1_R and AT_2_R) [10]. Ang II exhibits a higher affinity to the widely expressed AT_1_R whereby it exerts its main physiological effects by constricting blood vessels, increasing BP, and stimulating aldosterone release from adrenal glands, promoting water and salt reabsorption in the kidneys, thus, raising blood volume levels [10]. In the last decade, an ‘alternative axis’ has been identified involving the monocarboxypeptidase, ACE2, the biologically active peptide Ang-(1–7) and its G-protein coupled receptor, Mas (MasR). Ang-(1–7) is formed by the direct actions of ACE2 on Ang II or via ACE2 induced cleavage of Ang I, generating the nine amino acid peptide Ang-(1–9), which is further converted to Ang (1–7) by ACE or peptidases such as neprilysin (NEP) [8].

All the components of the ‘classical axis’ (angiotensinogen, renin, ACE, Ang II) have been identified within the brain parenchyma (see reviews [8, 11]). In addition, there is evidence that the ‘alternative axis’ is locally produced within the brain as ACE2 has been localised in neurons [12], astrocytes [13] and within the cerebrovasculature [14]. Similarly, the receptor subtypes responsible for mediating the functional effects of RAS peptides are expressed in neuronal and glial cells throughout the brain. For example, AT_1_ and AT_2_ receptors have been shown to be present in dopaminergic neurons, astrocytes and microglia from both human and primate brain tissue [15] and MasR shown to be expressed in neurons, astrocytes, microglia and cerebral endothelial cells in rodents [16, 17].

### Involvement in ischaemic stroke

Over-activation of the ACE/Ang II/AT_1_R axis is thought to contribute to the pathogenesis of AIS through its vasoconstrictor effects on cerebral vessels as well as its pro-inflammatory, pro-fibrotic and increased oxidative stress effects in the parenchyma [8]. For instance, in the brain, AT_1_R knockout (KO) mice subjected to permanent middle cerebral artery occlusion (pMCAO) exhibit a larger metabolic penumbra volume (cerebral protein synthesis and ATP mismatch) and higher CBF within the core and penumbra when compared to wild type (WT) mice whereas mice over-expressing human renin and angiotensinogen genes have larger infarcts [18, 19]. Furthermore, Ang II has been shown to enhance the contractile response in isolated middle cerebral arteries (MCA) following MCAO via the AT_1_R [20], therefore, worsening cerebral perfusion following ischaemia.

Interestingly, emerging evidence identifies the ‘alternative axis’ as an endogenous protective system, which acts to counteract the effects of the ‘classical axis’ by mediating vasodilation and anti-inflammatory, anti-oxidant and anti-apoptotic effects via MasR and AT_2_R activation [21, 22]. Following focal cerebral ischaemia in the rat, AT_2_Rs have been shown to be upregulated in the peri-infarct region of the cortex, and also within the selectively vulnerable regions of the cortex and hippocampus following global cerebral ischaemia [23, 24]. AT_2_R KO mice exhibit less ischaemic damage compared to WT control animals following MCAO [25] suggesting a protective role for this receptor in the setting of cerebral ischaemia. In support of a role for this pathway following acute ischaemic stroke the ACE2/Ang-(1–7)/Mas axis has been shown to be upregulated. Brain expression (mRNA and protein levels) of both ACE2 and MasR are upregulated from as early as 6 h following permanent MCAO in the rat, peaking at 24 h post MCAO. This was associated with an concomitant increase in both circulating serum and cerebral Ang-(1–7) levels [16]. This increase occurs during the first critical hours after stroke when ischaemic damage and loss of potentially salvageable penumbra is taking place, suggesting a potential role for this pathway in the pathogenesis. Increasing exogenous Ang-(1–7) levels in the brain by central infusion prior to stroke has been shown to decrease infarct volume in rats [26], providing evidence of a protective role for this pathway post-stroke.

The current evidence suggests that there is an imbalance in the RAS following stroke with an enhanced activation of the ACE/Ang II/AT_1_R pathway and that targeting the counter-regulatory ACE2/Ang-(1–7)/Mas axis may provide protection. This imbalance in the RAS may be exacerbated in the presence of known stroke risk factors such as gender and hypertension and therefore provide a potential therapeutic target in a subset of patients.

### RAS and stroke co-morbidities

Men have a higher incidence of stroke, however, after the menopause the incidence of stroke in women rapidly increases and this is co-incident with the decrease in female sex hormones [27]. In addition, to sex differences in stroke incidence, it is also well established in experimental stroke studies that female animals have less ischaemic injury than their male counterparts and that this protection is lost after ovariectomy [28, 29]. Furthermore, cells exhibit sex specific differences in the specific mechanisms of cell death following cerebral ischaemia [30] therefore it is not unlikely that sex differences will exist in the RAS in the brain and elsewhere. It is widely accepted that the RAS is influenced by sex hormones with males and females exhibiting differential responses to stimulation and inhibition of the RAS [31]. Differences in vascular reactivity to Ang II and receptor expression have been shown to be influenced by sex hormones, with testosterone stimulating the ‘classical axis’ and oestrogen lowering the AT_1_R:AT_2_R ratio, thereby enhancing vasodilatation [32–34]. Interestingly, in experimental animal models, female rats exhibit enhanced activity of the ACE2/Ang-(1–7)/Mas axis compared to males [31], showing increased renal Ang-(1–7) levels [35]. These findings may translate to humans, where normotensive healthy adult females have been shown to have higher plasma levels of Ang-(1–7) than their male counterparts [36]. These combined findings suggest that oestrogen may confer some degree of protection against stroke in premenopausal women by promoting increased activation of the ACE2/Ang-(1–7)/Mas axis and therefore, the loss of oestrogen associated with menopause may contribute to increased stroke risk through a loss of this enhanced protective pathway. In terms of brain expression, AT_1_R expression has been shown to be lowered in rats treated with oestrogen plus ovariectomy compared to ovariectomy alone. In contrast, oestrogen treatment in ovariectomised rats resulted in an increased expression of the AT_2_R compared to ovariectomised rats [37]. Similarly, mRNA expression of the AT_1_R and ACE are increased in the brains of hypertensive ovariectomised rats compared to intact females [38].

Hypertension is the single most important modifiable risk factor for the development of stroke, clinical outcome is poorer in patients with hypertension during acute stroke and in experimental animal models of stroke, ischaemic damage is significantly increased in hypertensive animals [39, 40]. Dysregulation of the RAS has been implicated in the development of hypertension, where hyperactivity of Ang II and other RAS components lead to enhanced oxidative stress and inflammation. Preclinical models of genetic hypertension have demonstrated increased AT_1_R expression in the vasculature of spontaneously hypertensive rats (SHR) compared to age-matched normotensive controls [41]. In addition to hyperactivity of the ‘classical axis’, dampening of the protective counter-regulatory axis is also evident, where hypertensive rat strains exhibit decreased ACE2 mRNA and protein expression compared to normotensive controls [42]. Chronic treatment of diabetic SHR rats with either an AT_1_R blocker (olmesartan) or ACE inhibitor (enalapril) reverses the microcirculatory changes that occur in pial vessels (functional and structural rarefaction) of the brain resulting in an improved cerebral perfusion and reduced cerebral oxidative stress [43]. Candesartan treatment in salt loaded SHRSP rats was shown to increase endothelial cell progenitor (EPC) colony number and reduce oxidative stress levels in mononuclear cells. This study suggests that ARB treatment may also act to improve endothelial cell function and angiogenesis in the presence of hypertension [44]. These results demonstrate the influence of the ACE/Ang II/AT1R pathway on remodelling of the cerebral microvasculature and suggest that overactivation of this pathway may contribute to the pathology.

Therefore, manipulation of the RAS towards the protective ACE2/Ang-(1–7)/MasR pathway in the presence of co-morbidities may shift the balance to prevent the exacerbation of ischaemic damage following AIS.

### Therapeutic targeting of the RAS following stroke

#### AT_1_R blockers

Angiotensin type 1 receptor blockers (ARBs or “sartans”) have been widely used as a successful and established therapy for the treatment of clinical hypertension [45]. As a result, this class of drug has been assessed for possible neuroprotective effects in a number of experimental stroke studies (Table 1) [46–56]. Central administration of irbesartan (ARB) prior to transient middle cerebral artery occlusion (tMCAO) has been shown to improve neurological outcome with no effect on blood pressure, however one limitation was the lack of any measure of infarct volume [43]. Follow up studies demonstrated that administration of ARB’s prior to MCAO could in fact reduce infarct volume and this was associated with a reduction in the number of activated microglia and macrophages (ED-1 staining) as well as a reduction in markers of apoptosis (TUNEL, PARP P85 staining and caspase 3) [47, 56].

**Table 1:** Experimental stroke studies using AT_1_ receptor antagonists

Animals Gender Strain Weight	Stroke model	*Treatment profile* AT_1_R blocker Administration Dose Time point	In vivo measures and methods	Treatment outcome	Proposed underlying mechanism	Reference
Male Wistar rats 250 g	*tMCAO* 90 min 24 h recovery	***Irbesartan*** i.c.v infusion or injection 0.5, 2.0, 5 nmol injection or 2 nmol/h infusion *Pre and post treatment*	***BP***: pressure transducer ***NS***: Bederson score ***Drinking response***	*Low dose treatment* Did ***not affect*** BP ***Improved*** NS ***Abolished*** Ang II induced drinking response	Decreased c-Fos and c-Jun protein expression in ipsilateral cerebral cortex	Dai et al. [46]
Male Wistar rats 200 g	*tMCAO* 90 min 3 or 7 days recovery	***Irbesartan*** i.c.v infusion 2 nmol/h *Pre and post treatment*	***BP***: pressure transducer ***NS***: Bederson and Garcia scores ***CBF***: laser Doppler ***Infarct volume***: cresyl violet staining and quantitative histopathology	Did ***not affect*** BP Did ***not affect*** CBF ***Improved*** NS ***Decreased*** infarct volume	***Anti-inflammatory and anti-apoptotic*** Decrease in TUNEL, PARP positive cells and activated microglia (ED-1 marker) in cortical peri-infarct areas	Lou et al. [47]
Male Wistar rats 280–305 g	*tMCAO* 3 h 24 h recovery	***Candesartan*** i.v bolus 1 mg/kg *Post treatment*	***BP***: telemetry method ***NS***: Bederson score ***Infarct volume***: TTC ***Cerebral oedema***: hemisphere volume analysis ***Haemoglobin content***	***Improved*** NS ***Decreased*** BP ***Decreased*** infarct volume ***Decreased*** cerebral oedema ***Decreased*** haemoglobin content	Not discussed	Fagan et al. [48]
Male Wistar rats 120–130 g	*tMCAO* 2 h 24 h recovery	***Candesartan*** 0.5 or 1 mg/kg i.p bolus *Pre-treatment*	***BP***: telemetry method ***Infarct volume***: TTC	***Decreased*** BP ***Decreased*** infarct volume	***Anti-oxidant and pro-regenerative*** Decrease in HIF-α and 8-OHdG positive cells and upregulation of eNOS and growth associated proteins, MAP-2, GAP-43 and cyclin D1	Liu et al. [49]
Male Wistar rats 160–200 g	*tMCAO* 90 min 48 h recovery	***Candesartan*** s.c bolus 0.1 mg/kg *Pre-treatment*	***BP***: pressure transducer ***NS***: Garcia score ***CBF***: laser-Doppler ***Infarct volume***: MRI T_2_ scan	Did ***not affect*** BP Did ***not affect*** CBF ***Improved*** NS ***Decreased*** infarct volume	***Activation of BDNF/TrkB signalling pathway*** Upregulation of BDNF gene expression and TrkB neurotrophin receptor protein levels in infarct and penumbral areas	Krikov et al. [50]
Male Sprague–Dawley rats 180–250 g	*tMCAO* 2 h 7 day recovery	***Olmesartan*** i.p infusion 0.001, 0.01, 0.1 or 1 μmol/kg/h *Post treatment*	***BP***: tail cuff method ***NS***: 34 point score ***Infarct volume***: TTC ***Cerebral oedema***: microgravimetry	*Treatment at low dose* Did ***not affect*** BP ***Improved*** NS ***Decreased*** infarct volume ***Decreased*** cerebral oedema	Downregulation of Ang II, MMP-2, MMP-9 and MT1-MMP protein levels in ischaemic area	Hosomi et al. [51]
Male Sprague–Dawley rats 250–275 g	*tMCAO* ET-1 induced MCAO 48 h recovery	***Candesartan*** s.c infusion 0.2/mg/kg per day *Pre-treatment*	***BP***: telemetry method ***NS***: Bederson and Garcia scores ***Behavioural testing (BHT)***: seed eating test ***Infarct volume***: TTC	Did ***not affect*** BP ***Improved*** motor function ***Improved*** NS ***Decreased*** infarct volume	Not discussed	Mecca et al. [52]
Male Sprague–Dawley rats 200–220 g	*tMCAO* 60 min Up to 28 days recovery	***Fimasartan*** Oral administration 0.5, 1 or 3 mg/kg *Pretreatment*	***BP***: CODA noninvasive BP system ***BHT***: limb placing test ***Infarct volume***: Nissl staining and TTC	*Treatment at low dose* Did ***not affect*** BP ***Improved*** functional recovery ***Decreased*** infarct volume	***Anti-inflammatory*** Attenuation of activated microglia (Ox6 staining), IκB degradation and COX-2 expression in peri infarct areas	Kim et al. [53]
Male SHR 270–306 g	*tMCAO* 60–120 min 24 h recovery	***Candesartan*** s.c infusion 0.5 mg/kg per day *Pre-treatment*	***BP***: tail cuff method ***CBF***: laser-Doppler ***Infarct volume***: TTC ***Cerebral oedema***	Did ***not affect*** BP compared to WKY rats ***Improved*** CBF ***Decreased*** infarct volume ***Decreased*** cerebral oedema	***Normalised autoregulation*** Decrease in AT_1_R protein expression in the nucleus of the solitary tract and area postrema	Nishimura et al. [54]
Male SHR 190–240 g	*pMCAO* dMCAO model 24 h recovery	***Candesartan*** s.c infusion 0.1 or 0.3 mg/kg per day *Pretreatment*	***BP***: tail cuff method ***CBF***: autoradiography ***Infarct volume***: TTC ***Cerebral oedema***	***Improved*** CBF ***Decreased*** BP ***Decreased*** infarct volume ***Decreased*** cerebral oedema	***Normalised autoregulation*** Attenuation of MCA media thickness	Ito et al. [55]
Male C57BL/6 mice 20 g	*pMCAO* 24 h recovery	Valsartan i.p infusion 3 mg/kg per day *Pre-treatment*	BP: method not specified NS: Bederson score CBF: laser-Doppler Infarct volume: TTC	Did not affect BP Improved NS Improved CBF Decreased infarct volume	Anti-oxidant and pro-angiogenic Decrease in MCP-1, TNF-α gene expression and superoxide levels and an increase in eNOS, NO and capillary density markers (PECAM-1; Glut-1)	Li et al. [56]

Studies involved either transient middle cerebral artery (tMCAO) or permanent middle cerebral artery occlusion (pMCAO). Unless specified, tMCAO was performed via intraluminal filament model

*8-OHdG* 8-hydroxy-2′-deoxyguanosine, *Ang II* angiotensin II, *AT*_*1*_ *R* angiotensin II type I receptor, *BDNF* brain derived neurotrophic factor, *BHT* behavioural testing, *BP* blood pressure, *CBF* cerebral blood flow, *COX-2* cyclooxygenase 2, *dMCAO* distal middle cerebral artery occlusion model, *ED-1* anti cluster differentiation 68 antibody, *eNOS* endothelial nitric oxide synthase, *ET-1* endothelin-1, *GAP-43* growth associated protein 43, *Glut-1* glucose transporter 1, *HIF-α* hypoxia inducible factor alpha, *i.c.v* intracerebroventricular, *IkB* IkappaB, *i.p* intraperitoneal, *i.v* intravenous, *MAP-2* microtubule-associated protein 2, *MCA* middle cerebral artery, *MCP-1* macrophage chemokine protein 1, *MMP* matrix metalloproteinase type 2, *MMP-9* matrix metalloproteinase type 9, *MRI* magnetic resonance imaging, *MT1-MMP* membrane type 1 matrix metalloproteinase, *NO* nitric oxide, *NS* neurological score, *PARP* poly(ADP-ribose) polymerase, *PECAM-1* platelet endothelial cell adhesion molecule 1, *SHR* spontaneously hypertensive rats, *TNF-α* tumor necrosis factor alpha, *s.c* subcutaneous, *TrkB* tropomyosin receptor kinase B, *TTC* 2,3,5-triphenyltetrazolium chloride staining, *TUNEL* terminal deoxynucleotidyl transferase dUTP nick end labelling

The cerebral vasodilatory potential of ARB’s have been extensively investigated and studies have examined its effects on isolated cerebral vessels as well as the cerebral blood flow (CBF) response in vivo. Candesartan in particular, has shown the potential to increase cerebral perfusion following MCAO in both normotensive as well as hypertensive rats (SHR). In normotensive rats, administration of Candesartan as an i.v bolus (2 h prior to MCAO) was reported to increase CBF in the ipsilateral hemisphere both at baseline and during MCAO [57]. In SHRs, chronic candesartan infusion for 28 days prior to MCAO reduced infarct volume and this was associated with an improved CBF compared to vehicle treated rats, particularly in the cortical areas at the periphery of the infarct. In addition, isolated vessels taken at the end of the chronic treatment protocol demonstrated an increased MCA diameter and reduced media thickness suggesting chronic changes to cerebral vessels resulting in reduced hypertension induced remodelling and enhanced collateral flow [55]. Similarly, Nishimura and colleagues demonstrated that chronic pre-treatment with Candesartan improved cerebrovascular autoregulation and decreased infarct size, an outcome associated with reduced AT_1_R binding in the MCA with Ang II autoradiography [54].

Other reported effects of AT_1_R blockade include pro-angiogenic/neurogenic effects. For example, Candesartan pre-treatment (at a dose with no BP effect) reduced infarct volume and increased mRNA expression of brain derived neurotrophic factor (BDNF) and its associated receptor (tropomyosin receptor kinase B; TrkB) 48 h after tMCAO [46]. Similarly, in SHR rats candesartan treatment following MCAO was shown to increase BDNF protein levels [58], suggesting, a potential involvement in neuronal cell regeneration. Despite indications of neuroprotection with blockade of the AT_1_R, not all experimental studies attenuated infarct evolution independently of BP lowering effects (Table 1).

#### AT_2_R agonism

It has been proposed that the neuroprotective mechanisms induced by ARB’s may partly involve increased Ang II binding to the AT_2_ receptor [59]. Consequently, selective AT_2_R agonists have been developed and investigated in models of experimental stroke (Table 2) [60–67]. In normotensive rats, central and systemic administration of compound 21 (C21), a selective non-peptide and orally active AT_2_R agonist, prior to and post ET-1 induced MCAO, reduces infarct size and improves neurological deficit. This protective effect was attributed to a decrease in inflammatory markers, inducible nitric oxide synthase (iNOS) and C–C motif chemokine receptor type 2 (CCR2) mRNA expression in the cerebral cortex following tMCAO, an effect blocked by the AT_2_R selective antagonist PD123319 [62].

**Table 2:** Experimental stroke studies using AT_2_ receptor agonists

Animals Gender Strain Weight	Stroke model	*Treatment profile* AT_2_R agonist Administration Dose Time point	In vivo measures and methods	Treatment outcome	Proposed underlying mechanism	Reference
Male Wistar rats 280–320 g	*tMCAO* 90 min or 3 h 24 h or 7 day recovery	***C2*** **1** i.p bolus 0.03 mg/kg *Post treatment*	***BP***: telemetry method ***NS***: Bederson score ***BHT***: Beam walk, paw grasp, rotarod test, grip strength ***Infarct volume***: TTC ***Haemoglobin content***	Did ***not affect*** BP ***Improved*** NS ***Improved*** functional outcome ***Decreased*** infarct volume ***Decreased*** haemorrhage	***Pro-angiogenic*** Via Akt/eNOS/NO pathway Upregulation of p-Akt, IL-10, BDNF and eNOS protein expression. Plus, nitrative stress markers nitrotyrosine and iNOS protein expression were downregulated in the ipsilateral hemisphere The results were further correlated to a decrease in AT_1_R and an upregulation of AT_2_R cerebral expression	Alhusban et al. [60]
Male Wistar rats 250–310 g	*tMCAO or pMCAO* Filament model Up to 21 days recovery	***C21*** i.p bolus 0.3 mg/kg/day *Post treatment*	***NS***: 7 point score ***Infarct volume***: Nissl staining	For pMCAO treated rats ***Improved*** NS ***Decreased*** infarct volume	***Pro-angiogenic*** Increased VEGF expression due to Akt/mTOR signalling pathway activation	Mateos et al. [61]
Male Sprague–Dawley rats 250–275 g	*tMCAO* ET-1 model 3 day recovery	***C21*** i.c.v or i.p infusion 0.0075 μg/μl/h i.c.v 0.03 or 0.1 mg/kg i.p *Pre and post treatment*	***BP***: tail cuff method ***CBF***: laser-Doppler ***Infarct volume***: TTC ***NS***: Bederson and Garcia scores	Did ***not affect*** BP Did ***not affect*** CBF ***Improved*** NS ***Decreased*** infarct volume	***Anti-inflammatory*** Decrease in gene expression for inflammatory markers iNOS, CCR2 and its ligand CCL2 in ipsilateral cerebral cortex	Joseph et al. [62]
Male SHR 270–320 g	*tMCAO* ET-1 model 3 day recovery	***CGP42112*** i.c.v infusion 0.1–10 ng/kg/min *Pre and post treatment*	***BP***: tail cuff method ***Infarct volume***: ballistic light method ***BHT***: ledged beam test	Did ***not affect*** BP ***Improved*** motor function ***Decreased*** infarct volume	***Anti-oxidant*** Decreased superoxide production in infarcted cortical regions, associated to an increase in brain AT_2_R expression	McCarthy et al. [63]
Male SHR Weight not specified	*tMCAO* ET-1 model 3 day recovery	***CGP42112*** i.c.v injection 3 μg/kg *Post treatment*	***BP***: tail cuff method ***Infarct volume***: ballistic light method ***BHT***: ledged beam test	Did ***not affect*** BP ***Improved*** motor function ***Decreased*** infarct volume	***Anti-apoptotic*** Decreased cleaved caspase-3 positive apoptotic cells and increased neuronal survival (NeuN positive cells) in ipsilateral hemisphere. Plus, increased activated microglia (OX42 marker) in ipsilateral core	McCarthy et al. [64]
Male SHR 330–350 g	*tMCAO* ET-1 model 3 day recovery	***C21*** i.c.v infusion and injection 3 μg/kg *Pre and post treatment*	***BP***: tail cuff method ***Infarct volume***: ballistic light method ***BHT***: ledged beam test	Did ***not affect*** BP ***Improved*** motor function ***Decreased*** infarct volume	***Anti-apoptotic and vasodilatory*** Increased neuronal survival (NeuN positive cells) and activated microglia which are potentially BDNF positive Myography studies in basilar arteries further suggested a vasodilatory effect induced by C21	McCarthy et al. [65]
Male C57BL/6J 8–12 weeks	*tMCAO* 30 min 24 h recovery	***CGP42112*** i.p bolus 1 mg/kg *Post treatment*	***CBF***: laser-Doppler ***NS***: Bederson score ***BHT***: hanging wire test ***Infarct volume***: thionin staining ***Cerebral oedema***	Did ***not affect*** cerebral oedema ***Improved*** NS ***Improved*** motor function ***Improved*** CBF ***Decreased*** infarct volume	***Anti-apoptotic*** C21 promotes cell viability in primary cortical neurons following oxygen glucose depravation challenge	Lee et al. [66]
Male C57BL/6J WT and AT_2_R KO mice 25–30 g	*pMCAO* dMCAO model 24 h recovery	***C21*** i.p bolus 10 µg/kg/day *Pre and post treatment*	***BP***: tail cuff method ***NS***: 4 point score **CBF**: laser speckle method Infarct volume: ***MRI*** T_2_ scan Cerebral oedema Blood brain barrier (BBB) permeability: Evans blue dye	Did ***not affect ***BP ***Improved*** NS ***Improved*** CBF ***Decreased*** infarct volume ***Decreased*** oedema ***Decreased*** BBB permeability	Ant-inflammatory Decreased expression of MCP-1, TNF-α and SO Also observed reduced BBB breakdown	Min et al. [67]

Studies involved either transient middle cerebral artery (tMCAO) or permanent middle cerebral artery occlusion (pMCAO). Unless specified, tMCAO was performed via intraluminal filament model

*Akt* protein kinase B, *AT*_*1*_ *R* angiotensin II type I receptor, *AT*_*2*_ *R* angiotensin II type II receptor, *BBB* blood brain barrier, *BDNF* brain derived neurotrophic factor, *BHT* behavioural testing, *BP* blood pressure, *C21* compound 21, *CBF* cerebral blood flow, *CCL2* chemokine (C–C motif) ligand 2, *CCR2* C–C chemokine receptor type 2, *COX-2* cyclooxygenase 2, *dMCAO* distal middle cerebral artery occlusion model, *eNOS* endothelial nitric oxide synthase, *ET-1* endothelin-1, *i.c.v* intracerebroventricular, *IL-10* interleukin 10, *iNOS* inducible nitric oxide synthase, *i.p* intraperitoneally, *MCP-1* macrophage chemokine protein 1, *MRI* magnetic resonance imaging, *mTOR* mechanistic target of rapamycin, *NeuN* neuronal nuclei, *NO* nitric oxide, *NS* neurological score, *OX-42* anti-CD11b/c antibody, *p-Akt* phosphorylated Akt, *SHR* spontaneously hypertensive rats, *SO* superoxide, *TNF-α* tumor necrosis factor alpha, *TrkB* tropomyosin receptor kinase B, *TTC* 2,3,5-triphenyltetrazolium chloride staining, *VEGF* vascular endothelial growth factor

In conscious SHR rats, the AT_2_R agonist, CGP42112, attenuates lesion progression and improves motor function following tMCAO. These effects were independent of BP alterations and possibly due to enhanced AT_2_R receptor level expression, increased microglial activation and reduced superoxide production within the peri-infarct region [63, 64]. These findings are supported by in vitro data in primary cortical neurons where CGP42112 administration was shown to attenuate cell death following oxygen glucose depravation (OGD) [66]. Similarly, C21, was shown to dose dependently reduce infarct volume in SHR when administered centrally for 5 days prior to endothelin 1 (ET-1) induced MCAO. This was associated with an increase in microglia activation within the infarct core and peri-infarct, however, when administered 6 h post-stroke the protective effect was still observed but there was no enhancement of microglial activation [65].

Apart from an anti-inflammatory role, C21 has also been shown to promote angiogenesis. In primary cortical neurones, C21 treatment 24 h post OGD challenge, enhances vascular endothelial growth factor (VEGF) via mechanistic targeting of rapamycin (mTOR) pathway activation [68]. Similarly, in in vivo models, 28 day C21 treatment in mice subjected to either transient or permanent MCAO resulted in increased angiogenesis via VEGF upregulation and effect which the authors hypothesise is through an AT_2_R mediated activation of the P13K-Akt-mTOR pathway [61]. Additionally, in ex vivo studies, vasodilation and increased perfusion seems to be dependent on the animal model used. Using wire myography, C21 treatment in isolated basilar arteries causes cerebral vessel relaxation, promoting vasodilation [59], an outcome supported by in vivo studies where mice subjected to pMCAO with C21 pre-treatment had an improved CBF in the ischaemic hemisphere at days 1 and 3 following pMCAO. This was associated with a decreased infarct size, and attenuated blood brain barrier (BBB) breakdown as measured by Evans blue extravasation [67]. On the contrary, in mice subjected to tMCAO, C21 did not induce any acute changes in CBF when administered following reperfusion [68].

In contrast to the protective effects of C21 discussed above, a recent study showed that C21 did not affect infarct volume in mice 4 days following tMCAO; however, it did improve neurological score and mortality rates. Interestingly, the neurological improvement observed was associated with an increase in anti-apoptotic and regenerative molecules BDNF, TrkB and growth associated protein 43 (GAP-43) in peri-infarct regions when compared to vehicle [68].

#### MasR agonism

Evidence suggests that AT_2_R interacts with other RAS mediators and its effects may be partly mediated by an interaction with the Mas receptor, which is activated by Ang-(1–7) [69]. Recently, the interaction between ACE2/Ang-(1–7)/MasR in AIS has been studied and shown to be protective in several animal models (Table 3) [17, 26, 70–73]. Mecca and colleagues were one of the first groups to identify the potential neuroprotective effects of this peptide and its receptor. They demonstrated that central administration of Ang-(1–7) for 7 days prior to ET-1 induced MCAO, reduced infarct volume via MasR activation and decreased cortical iNOS mRNA expression [26]. Further evidence now proposes that Ang-(1–7) might have direct anti-inflammatory properties and specifically target microglia. In primary microglial cell cultures under basal conditions, MasR activation modulates inflammatory marker expression by attenuating pro-inflammatory genes [74]. Following tMCAO injury, Ang-(1–7) treatment not only reduces iNOS mRNA and protein levels in ipsilateral cerebral cortex but attenuates chemokine C-X-C motif ligand 12 (CXCL12) levels at 6 h post tMCAO and interleukin (IL)-1β, IL-6 and cluster differentiation 11b (CD11b) at 24 h [17]. These findings are further supported by pMCAO studies, where Ang-(1–7) decreased nuclear factor kappa B (NFκB) phosphorylation and cyclooxygenase-2 (COX-2) protein levels in peri-infarct regions when compared to vehicle [71].

**Table 3:** Experimental stroke studies using Mas receptor agonists

Animals Gender Strain Weight	Stroke model	*Treatment profile* MasR agonist Administration Time point	In vivo measures and methods	Treatment outcome	Proposed underlying mechanism	Reference
Male Sprague–Dawley rats 280–320 g	*tMCAO* 90 min Up to 72 h recovery	***Ang-(1–7)*** i.c.v infusion 1 pmol/0.5 µl/h, 100 pmol/0.5 µl/h or 10 nmol/0.5 µl/h *Post treatment*	***NS***: 6 point score	***Improved*** NS	Increased NO and eNOS expression in ischaemic core and penumbral areas	Zhang et al. [70]
Male Sprague–Dawley rats 250–275 g	*tMCAO* ET-1 model 3 day recovery	***Ang-(1–7)*** i.c.v infusion 1.1 nM; 0.5 μl/h *Pre and post treatment*	***BP***: tail cuff **CBF**: laser-Doppler ***Infarct volume***: TTC ***NS***: Bederson and Garcia scores ***BHT***: seed eating test	Did ***not affect*** BP or CBF Did ***not affect*** CBF ***Improved*** NS ***Improved*** motor function ***Decreased*** infarct volume	Decreased iNOS levels in ipsilateral hemisphere	Mecca et al. [26]
Male Sprague–Dawley rats 250–280 g	*pMCAO* Filament model 24 h	***Ang-(1–7)*** i.c.v infusion 1.11 nM; 1 μl/h *Pre and post treatment*	***CBF***: laser-Doppler ***Infarct volume***: TTC **NS**: Bederson score	Did ***not affect*** CBF ***Improved*** NS ***Decreased*** infarct volume	***Anti-oxidant and anti-inflammatory*** Decrease in oxidative stress marker, malondialdehyde and increased SOD activity Reduced expression of inflammatory markers NFκB, COX-2, TNF-α and IL-1β	Jiang et al. [71]
Male Sprague–Dawley rats 250–275 g	*tMCAO* ET-1 model Up to 24 h recovery	***Ang-(1–7)*** 1.1 nM; 0.5 μl/h i.c.v infusion *Pre and post treatment*	***Infarct volume***: TTC	***Decreased*** infarct volume	***Anti-inflammatory*** Decrease in IL-1α, IL-6, CXCR4 as well as iNOS and microglia marker CD11b expression in ipsilateral cortex	Regenhardt et al. [17]
Male Sprague–Dawley rats 250–280 g	*pMCAO* Filament model 24 h recovery	***Ang-(1–7*** **)** i.c.v infusion 1.1 nM; 0.25 μl/h *Pre-treatment*	***BP***: Tail cuff method ***CBF***: laser-Doppler ***NS***: Bederson score ***Infarct volume***: TTC	Did ***not affect*** BP ***Improved*** NS ***Improved*** CBF ***Decreased*** infarct volume	***Pro-angiogenic*** Increase in NO, eNOS and VEGF protein levels and capillary density markers CD31 in ipsilateral hemisphere	Jiang et al. [72]
Male C57BL6/J mice 22–30 g	*tMCAO* 60 min 24 h recovery	***AVE0991*** i.p bolus 20 mg/kg *Post treatment*	***CBF***: laser-Doppler ***NS***: Bederson score ***BHT***: Open field and parallel rod floor test ***Infarct volume***: Thionin staining	Did not affect CBF Did not affect NS Did not affect BHT Did not affect infarct volume	Not discussed	Lee et al. [73]

Studies involved either transient middle cerebral artery (tMCAO) or permanent middle cerebral artery occlusion (pMCAO). Unless specified, tMCAO was performed via intraluminal filament model

*Ang-(1–7)* angiotensin-(1–7), *BHT* behavioural testing, *BP* blood pressure, *C21* compound 21, *CBF* cerebral blood flow, *CD11b* cluster differentiation 11b, *CD31* cluster differentiation 31, *COX-2* cyclooxygenase-2, *CXCR4* C-X-C motif chemokine receptor type 4, *eNOS* endothelial nitric oxide synthase, *ET-1* endothelin-1, *i.c.v* intracerebroventricular, *IL-1α* interleukin 1 alpha, *IL-1β* interleukin 1 beta, *IL-6* interleukin 6, *iNOS* inducible nitric oxide synthase, *i.p* intraperitoneally, *MasR* Mas receptor, *NFκB* nuclear factor kappa B, *NO* nitric oxide, *NS* neurological score, *SOD* superoxide dismutase, *TNF-α* tumor necrosis factor alpha, *TTC* 2,3,5-triphenyltetrazolium chloride staining, *VEGF* vascular endothelial growth factor

The vasodilatory properties of Ang-(1–7) in cerebral vessels are conflicting. In canine MCA and piglet pial arterioles, Ang-(1–7) induces vasodilation in intact vessels only at very high concentrations [75, 76]. However, Durand and colleagues demonstrated that in normotensive isolated rat MCA's, Ang-(1–7) dose dependently induced a vasodilator response which was blocked by Mas and AT_2_ receptor antagonists [77]. Ang-(1–7) mediated vasodilatation may vary depending on animal species. Nevertheless, in rodents, it is possible that Ang-(1–7) might induce vasodilation in cerebral vessels due to increases in NO and/or bradykinin (BK) release. Central administration of Ang-(1–7) in a rat transient MCAO model has been shown to enhance nitric oxide (NO) release between 3 and 72 h after transient MCAO when compared to vehicle treated rats and this was associated with an increased mRNA and protein expression of endothelial nitric oxide synthase (eNOS) in ischaemic brain tissue at these acute time points following MCAO. In addition, concentration levels of BK and its receptors were shown to be upregulated in ischaemic cortex following Ang-(1–7) treatment between 6 and 48 h post tMCAO [70, 78].

Similarly, 4-week chronic Ang-(1–7) infusion prior to MCAO was shown to increase protein levels of eNOS and NO concentration in the ischaemic hemisphere whereas iNOS and neuronal (nNOS) expression were unchanged. Interestingly, brain VEGF protein levels were also elevated, an outcome associated with an increase in angiogenic markers (CD31 and EdU). Following pMCAO, Ang-(1–7) reduced infarct volume and improved CBF an effect specific to the MasR, suggesting that Ang-(1–7) activates Mas/eNOS signalling pathways, improving angiogenesis and cerebral perfusion [72]. Still, the impact of Ang-(1–7) on CBF is under debate as Mecca et al. [26] reported that central infusion of Ang-(1–7) for 7 days prior to ET-1 induced MCAO did not affect CBF when measured during MCAO.

Targeting the MasR with Ang-(1–7) is showing promising results in experimental stroke models however, Ang-(1–7) has a 20 s half life in the bloodstream and is unlikely to cross the BBB therefore necessitating specific receptor agonists to be developed with improved pharmacokinetic profiles [79]. As a result, in order to see an effect following AIS, it has had to be administered centrally, a route of administration which is not clinically feasible. At present, Mas agonist, AVE0991 has been developed, however, following tMCAO in mice, i.p AVE0991 post-treatment failed to induce similar neuroprotective as observed in Ang-(1–7) treated studies [73].

### The effect of ARBs in clinical trials

In clinical trials, the effectiveness of ARB treatment in preventing vascular events and mortality following AIS is under debate. The LIFE trial compared losartan (AT_1_R antagonist) and atenolol (selective β1 receptor blocker) treatment for preventing cerebral ischaemic events in patients with a clinical history of hypertension and left ventricular hypertrophy. After a follow up time of 4.8 years, the authors demonstrated that the use of losartan was associated with a decrease in the frequency of stroke [80]. Moreover the ACCESS trial was designed to assess the efficacy of a modest blood pressure reduction acutely after stroke (day 1 post stroke for 7 days) with patients receiving either candesartan cilexetil (AT_1_R antagonist) or placebo treatment. Although the trial was stopped early it did demonstrate a reduced number of vascular events and decreased mortality in the candesartan treatment group [81]. In following years, the MOSES study investigated the effects of eprosartan or nitrendipine (calcium channel blocker) in hypertensive patients who had a cerebrovascular event within the last 24 months prior to recruitment. After 2.5 years follow up, it was identified that eprosartan group showed significantly less cases of cardiovascular and cerebrovascular events [82]. Furthermore, telmisartan, as an alternative therapy for cardiovascular disease patients who are intolerant to ACE inhibitors, was shown to induce a modest reduction in stroke incidence [83].

Despite the benefits of AT_1_R antagonism observed, other studies showed contradictory effects. For instance, a study assessing whether blood pressure lowering with telmisartan treatment in ischaemic stroke patients affected the risk of recurrent cerebral events concluded after a 2.5 year follow up that telmisartan did not affect the risk of recurrent stroke nor other cardiovascular events [84]. More recently, the SCAST clinical trial investigated whether blood pressure lowering with candesartan in acute stroke patients is beneficial. After 6 months follow up, no beneficial effect of blood pressure lowering with candesartan was observed with no difference in the occurrence of vascular events. Plus, for functional outcome there was a less favourable modified Rankin score in the candesartan group albeit this was not statistically significant [85]. The results from clinical trials to date have primarily investigated the influence of modulation of the RAS in terms of stroke incidence or blood pressure lowering strategies following stroke however, the effect of RAS modulation acutely after ischaemic stroke in terms of outcome (i.e. penumbral salvage, lesion volume) has yet to be fully investigated.

## Conclusions

The RAS is currently a therapeutic target for the treatment of AIS. Pathological activation of the ‘classical axis’ contributes towards ischaemic injury development and the ‘alternative axis’ is thought to counteract these effects leading to protection. Interestingly, the pathology of stroke co-morbidities such as hypertension and gender are all influenced by RAS dysfunction, where there is an increased/overactive ACE/Ang II/AT_1_R pathway. Consequently, targeting the RAS could act as a preventive line of therapy for the development of stroke and attenuate injury following stroke by diminishing brain RAS imbalances or providing direct neuroprotection.

Several preclinical studies have identified the potential neuroprotective effect of AT_1_R blockers, AT_2_R and MasR agonists. ARBs have been extensively studied in the context of experimental stroke due to its current clinical use in hypertension management. Although there were indications of a level of neuroprotection induced by ARBs, its clinical potential has been set aside due to clinical trials that showed that ARBs may induce harmful effects in ischaemic stroke patients. On the other hand, AT_2_R and MasR agonism are showing promising effects in preclinical stroke models; however, further studies have to be conducted to identify the actual mechanisms induced following stroke, addressing several limitations associated to preclinical stroke.

Further understanding of the role of the RAS following ischaemic stroke, in particular the role of the ACE2/Ang-(1–7)/Mas pathway and development of improved pharmacological drugs targeting the central RAS components are needed before any successful translation would be possible.

## Abbreviations

ACE: angiotensin converting enzyme
ACE2: angiotensin converting enzyme 2
AIS: acute ischaemic stroke
ARB: angiotensin type 1 receptor blockers
AT1R: angiotensin type 1 receptor
AT2R: angiotensin type 2 receptor
BBB: blood brain barrier
BP: blood pressure
CBF: cerebral blood flow
C21: compound 21, AT2 receptor agonist
EPC: endothelial progenitor cell
KO: knockout
MasR: Mas receptor
pMCAO: permanent middle cerebral artery occlusion
tMCAO: transient middle cerebral artery occlusion
NEP: neprilysin
NO: nitric oxide
OGD: oxygen glucose deprivation
RAS: renin angiotensin system
SHR: spontaneously hypertensive rat
WT: wild type
VEGF: vascular endothelial growth factor
